# Robocalypse? Yes, Please! The Role of Robot Autonomy in the Development of Ambivalent Attitudes Towards Robots

**DOI:** 10.1007/s12369-021-00817-2

**Published:** 2021-08-13

**Authors:** Julia G. Stapels, Friederike Eyssel

**Affiliations:** Department of Psychology, Center for Cognitive Interaction Technology, Bielefeld University, Bielefeld, Germany

**Keywords:** Robot evaluation, Human–robot interaction, Attitudes towards robots, Ambivalence, Autonomy

## Abstract

Attitudes towards robots are not always unequivocally positive or negative: when attitudes encompass both strong positive and strong negative evaluations about an attitude object, people experience an unpleasant state of evaluative conflict, called ambivalence. To shed light on ambivalence towards robots, we conducted a mixed-methods experiment with *N* = 163 German university students that investigated the influence of robot autonomy on robot-related attitudes. With technological progress, robots become increasingly autonomous. We hypothesized that high levels of robot autonomy would increase both positive and negative robot-related evaluations, resulting in more attitudinal ambivalence. We experimentally manipulated robot autonomy through text vignettes and assessed objective ambivalence (i.e., the amount of reported conflicting thoughts and feelings) and subjective ambivalence (i.e., self-reported experienced conflict) towards the robot ‘VIVA’ using qualitative and quantitative measures. Autonomy did not impact objective ambivalence. However, subjective ambivalence was higher towards the robot high versus low in autonomy. Interestingly, this effect turned non-significant when controlling for individual differences in technology commitment. Qualitative results were categorized by two independent raters into assets (e.g., assistance, companionship) and risks (e.g., privacy/data security, social isolation). Taken together, the present research demonstrated that attitudes towards robots are indeed ambivalent and that this ambivalence might influence behavioral intentions towards robots. Moreover, the findings highlight the important role of technology commitment. Finally, qualitative results shed light on potential users’ concerns and aspirations. This way, these data provide useful insights into factors that facilitate human–robot research.

## Introduction

Are you afraid that your robotic companion will one day strive for world domination? Science fiction media have often portrayed a so-called ‘robot apocalypse’ e.g., in the ‘I, robot’ (2004), ‘Terminator’ (1984, latest sequel: 2019), or ‘Matrix’ (1999, next sequel: 2022) movies. Such movies reflect the high hopes and great dangers associated with modern robotic systems. Thereby, cinematic depictions of robots contribute to ambivalent attitudes towards robots, particularly towards autonomous robots. On the one hand, classic and contemporary movies portray autonomous robots as highly capable and intelligent. On the other hand, such movies depict robots as making unpredictable decisions beyond human control, as taking over the world, and aiming to destroy humanity. Such considerations clearly have an impact on attitudes towards robots and potential end users’ willingness to engage with robots in and outside of the laboratory [29, 57].

Complementing existing work on the media image of robots, recent research has likewise suggested that autonomy in robots elicits ambivalent responses. Robot autonomy is defined as ‘the extent to which a robot can sense its environment, plan based on that environment, and act upon that environment with the intent of reaching some task-specific goal (either given or created by the robot) without external control.’ ([6], p.3). For one, autonomous robots are associated with bringing relief by supporting users with chores and other undesirable everyday tasks [29], while at the same time threatening people’s feelings of safety and human uniqueness [57]. However, the impact of robot autonomy on attitudes towards social robots, and the nature and content of those attitudes have not yet been investigated systematically. Given the prospective technological advancements, robots eventually become more and more autonomous. In light of this fact, it is crucial to investigate the influence of perceived autonomy on robot-related attitudes. What people think of robots may depend on a robot’s degree of perceived or actual autonomy. Likewise, negative user attitudes might guide behavior towards robots, contributing to lower levels of acceptance of such novel technologies.

Consequently, the goals of the current research were threefold: First, we explored the experience of ambivalence in attitudes toward autonomous robots; second, we investigated the role of robot autonomy in the formation of attitudes towards robots; and third, we identified the specific positive and negative evaluations associated with autonomous robots.

### Related Work

#### Ambivalent Attitudes Towards Robots

In social psychological research, attitudes are defined as all evaluations about one object of thought. Naturally, attitudes can be held towards any construct, e.g., people or even things [10]. Unsurprisingly, people hold attitudes about robots as well. What is less clear, however, is the very nature of the evaluations of robots. Attitudes towards robots reflect information from many sources (e.g., from science fiction movies, the news, or personal encounters), expectations and fears, experiences and illusions [51]. Consequently, recent research has suggested that users’ attitudes towards robots cannot simply be characterized as only positive or only negative in valence. In fact, attitudes might be ambivalent [52]. Ambivalence is defined as the experience of both positive and negative evaluations about one and the same attitude object [31, 53]. Ambivalence has been investigated in many domains (e.g., food choices [45], online transactions [36], artificial intelligence [35]) and it is associated with distinct affective, behavioral, and cognitive consequences. Among these are the experience of negative affect [55], choice delay [2], or systematic information processing [16].

It is key to conduct attitude research on ambivalence in order to overcome methodological shortcomings rooted in traditional research on univalent attitudes. Previous work on this issue has demonstrated that indeed, measurement approaches directly impact the outcomes of the respective assessments. That is, ratings based on semantic differentials might be interpreted as neutral, but may well be ambivalent. This is due to the fact that the response format associated with semantic differentials might obscure the actual nature of the attitudes. To avoid this critical issue, positive and negative attitudes should be measured separately [31], since positivity and negativity in attitudes are partly independent [12]. Thompson and colleagues have proposed a formula to measure objective ambivalence that integrates both positive and negative aspects of attitudes as measured by separate items [53]. By using this formula, positive and negative evaluations that might lead to ambivalent attitudes can be uncovered. Accordingly, low values on both the positive and the negative item or a low value on one item and a high value on the other indicate low objective ambivalence (i.e., neutrality or univalence), while high values on both items indicate high objective ambivalence. This way, varying degrees of objective ambivalence may be differentiated and e.g., high values on both items would indicate higher ambivalence than medium values on both items.

Above and beyond, Priester and Petty have proposed to additionally measure the experience of ambivalence (i.e., subjective ambivalence) directly [39]. This might be relevant in the context of ambivalent attitudes, because subjective ambivalence is not automatically experienced due to the existence of opposing evaluations (i.e., objective ambivalence). Those evaluations need to be active and relevant to evoke feelings of conflict. The measure of subjective ambivalence is related to objective ambivalence, with high levels of opposing evaluations predicting the subjective feeling of conflict [46]. Taken together, previous ambivalence research has demonstrated the necessity to measure the positive and the negative sides of attitudes separately (i.e., objective ambivalence), assess evaluative conflict (i.e., subjective ambivalence) directly, and has provided the means to apply the methodology to new attitude research fields, such as robotics.

In the context of social robotics, qualitative results from previous research reflect the ambivalent nature of attitudes towards robots. For instance, participants report feeling torn between benefits (e.g., receiving assistance in everyday life) and challenges associated with having a robot at home (e.g.,the fear of being dependent of the robot) [24, 25]. Furthermore, previous quantitative research has experimentally compared how measurement methods would impact self-reported attitudes. Importantly, this work has shown that the respective self-reported attitudes varied as a function of measurement approach, so that ambivalent attitudes appeared to be neutral on bipolar evaluation items, despite resulting in objective and subjective ambivalence and heightened arousal [52]. Therefore, to capture the ambivalent nature of attitudes towards robots, the positive and negative sides of evaluations concerning robots should be measured separately, while providing participants with the means to express attitudinal conflict. Moreover, in the context of artificial intelligence, which also plays a key role in robotic systems, ambivalence has been shown to explain more variance in behavioral intentions than univalent attitudes alone [35]. This indicates that ambivalence does not necessarily imply a weak attitude, but rather strong evaluations that influence behavioral intentions. In light of this initial empirical evidence, it becomes clear that further research is needed to understand ambivalent attitudes towards robots, as well as the specific evaluation contents that cause ambivalence.

#### Robot Autonomy as a Source of Ambivalence

With increasing technological progress, robots bear the potential of becoming more and more autonomous. The current technological advancements reflect this increase in robot autonomy: Robots learn to navigate more autonomously (e.g., [14]), how to make autonomous behavior decisions concerning their interaction strategies (e.g., [50]), and they are taught how to engage autonomously in more and more meaningful conversations (e.g., [13]). We propose that attitudes towards robots are characterized as ambivalent, with increased robot autonomy amplifying this effect. With growing robot autonomy, both positive and negative evaluations of robots increase, resulting in ambivalence towards autonomous systems.

On the one hand, higher robot autonomy is associated with perceived robot intelligence [15], a reduction in workload, user friendliness, and adaptability [49]. On the other hand, robot autonomy is associated with people feeling threatened in their distinctiveness as humans and with regard to their human identity [22]. Subjectively, autonomous robots pose a threat to human physical safety, resulting in opposition to robotics research [57], possibly inspired by science-fiction media [29]. In terms of evaluation contents related to social relationships, autonomous robots may also evoke strong positive as well as strong negative evaluations. This is due to the fact that robots may autonomously communicate with humans, a capacity that enables them to serve as social companions. At the same time, the deployment of such companion robots might potentially reduce interpersonal interactions between humans by substituting them [11, 54]. Furthermore, Dang and Liu showed that ambivalence is higher towards mindful compared to mindless robots [18]. Here, mindful robots were defined as robots with the capability of experience, expression, and action planning, which corresponds closely to autonomy.

In sum, people seem to evaluate high robot autonomy as positive and negative at the same time, and this brings us to the assumption that highly autonomous robots evoke higher ambivalence than robots low in autonomy.

#### Influence of Individual Differences on Attitudinal Ambivalence

The degree of participants’ technology commitment, a construct that reflects users’ previous experience with technologies and their readiness to engage with them has been shown to influence robot-related attitudes [8, 40]. That is, higher technology commitment might lead to more positive evaluations of a robot and a higher willingness to interact with it [8]. Since technology commitment seems to explain large parts of variance in attitudes towards robots, we included this construct in the current research.

Another factor that could influence users’ attitudes toward robots is individual loneliness [20]. Previous research has investigated the benefits of robot use with elderly participants in care homes, who are especially prone to loneliness (e.g., [32, 42]). Social robots stimulated positive interactions [32] and decreased feelings of loneliness in users [42]. However, surprisingly, more recent research indicates that loneliness seems even more pronounced in young people, especially in males living in individualistic societies, e.g., in Europe or North America [3]. It has been empirically demonstrated that situational loneliness increases anthropomorphism and mind attribution to robots [20]. That is, lonely people see robots as more ‘human’, making them more suitable as potential interaction partners. Plausibly, loneliness could attenuate attitudinal conflict concerning robots by making them seem more human and the interaction with them seem more rewarding, while disregarding negative attitude aspects [20].

#### Qualitative Aspects of Attitudes towards Robots

By using qualitative measures, participants get the opportunity to express their thoughts on attitude objects through open-ended questions. Thereby, we can gain insights into the specific issues that contribute to positive, negative, or ambivalent attitudes towards an attitude object [29]. Above and beyond a merely quantitative approach, previous qualitative research on attitudes toward robots has revealed conflicting evaluations in attitudes towards robots. Crucially, such ambivalent attitudes would not have been captured by quantitative measures alone [24, 25]. Thus, we integrated a qualitative approach to measuring evaluations in addition to utilizing quantitative measures in the current experiment.

## The Present Research

The present mixed-methods experiment integrated quantitative and qualitative measures and assessed the effect of robot autonomy on participants’ ambivalence, attitudes towards robots, and attitude contents. Previous research has manipulated autonomy through experimentally framing robots in general as more or less autonomous [57], or by investigating autonomous versus tele-operated robots [15]. In the present research, we manipulated robot autonomy by using concrete, text-based scenarios of a specific robot, i.e., the newly developed robot ’VIVA’ by navel robotics

(https://www.navelrobotics.com/viva). We did so to facilitate participants’ imagination of having a robot at home.

It is key to explore the potential influence of perceived or actual robot autonomy on attitudes towards robots. Exploring the interplay between ambivalence and autonomy is yet under-researched, but highly relevant because robot autonomy might cause both more positive and more negative evaluations, facilitating ambivalent attitudes. Measurement approaches from social psychology can help identify actual ambivalence in attitudes towards robots. By raising awareness about potentially ambivalent user attitudes, interventions to reduce attitudinal ambivalence can be utilized to increase positive attitudes and the readiness to interact with social robots.

We hypothesized that attitudes towards the VIVA robot would be more ambivalent (as measured via self-reported objective ambivalence [Hypothesis 1] and subjective ambivalence [Hypothesis 2]) in the high autonomy condition compared to the low autonomy condition. We included technology commitment as a covariate in our analyses. In addition to attitudinal ambivalence, we assessed attitudes, evaluations, and behavioral intentions towards a social robot in more detail using measures of likeability, trust, and contact intentions towards the VIVA robot.

## Method

We manipulated robot autonomy (low vs. high autonomy) by using robot descriptions and text-based human–robot interaction scenarios based on the VIVA robot. The interaction scenarios were carefully pretested in a pilot study to reflect low versus high levels of robot autonomy. The pilot study and the main experiment were approved by the Ethics Committee of Bielefeld University (application no. 2019-044, January 18th, 2019). We report how we determined our sample size, all data exclusions, all manipulations, and all measures in this study and in the preregistration (https://aspredicted.org/7d9u9.pdf).

### Participants and Design

A convenience sample with 200 university students was recruited at Bielefeld University, Germany. They were invited to participate in a 10 to 15-min laboratory-based experiment for 2€ or course credit. Reimbursement was in line with departmental regulations which suggest a max. of 10 € per hour for participant reimbursement. As preregistered, we stopped data collection at 200 participants based on a power analysis, and excluded *n* = 37 participants who failed at least one of the attention checks, resulting in *n* = 163 valid cases (108 female, 54 male, 1 not specified, $M_{Age} = 22.13$, $SD_{Age} = 3.13$). Robot autonomy (low vs. high) was manipulated between participants and participants were randomly assigned to one of the two conditions.

### Experimental Manipulation

**Fig. 1:**
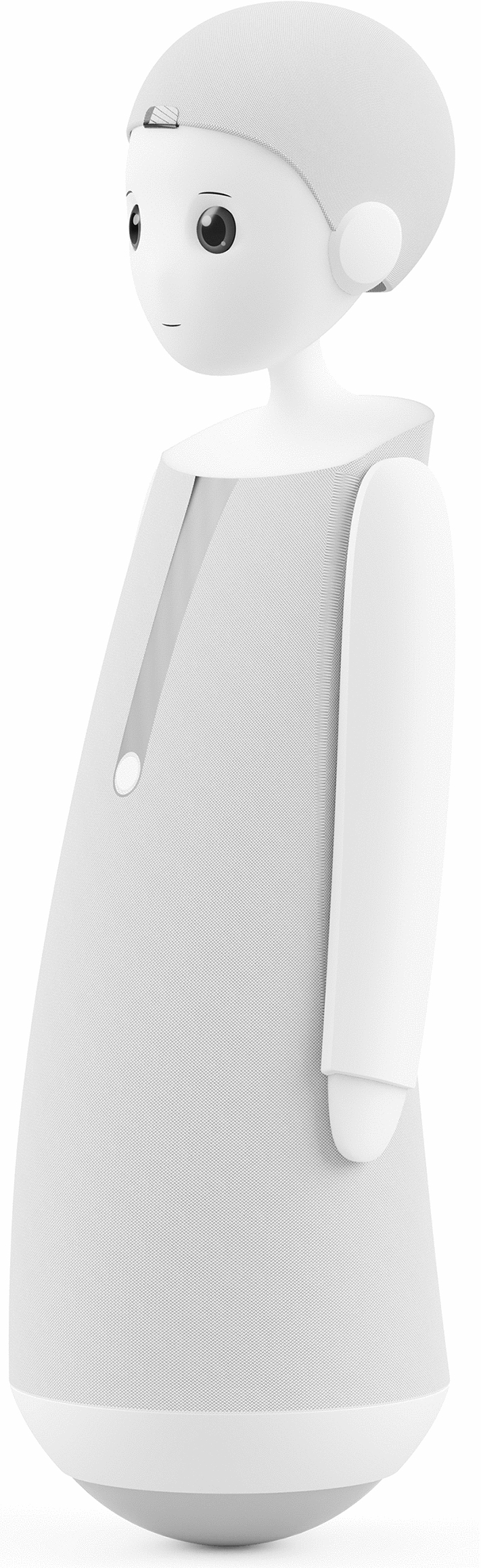
Design of the VIVA robot. Reprinted with permission by navel robotics GmbH

Initially, all participants were provided with a brief description and a picture of the VIVA robot (see Fig. 1). To manipulate autonomy, we created two versions, with information in brackets being different between conditions [low autonomy/ high autonomy]. The materials translate as follows:

VIVA is a social robot for everyone. VIVA has the height of an elementary school child and shows facial expressions through its animated eyes and mouth. VIVA’s body is mobile and suitable for social interactions. VIVA can roll and moves within the apartment [according to command/ independently]. [On demand/ Automatically and completely autonomously], VIVA captures the environment spatially through various sensors and video and audio systems, identifies people and objects [on request/ -], and understands speech. VIVA can [-/ ask and] answer questions and [list activities on demand/ autonomously propose and plan activities]. VIVA also has its own needs and, for example, drives to the charging station [on demand/ at its own discretion]. In addition, VIVA can carry out prompts, e.g., drive to a specific location, or reproduce information. Overall, VIVA is a new, [not very/ very] autonomous social lifestyle robot that will be on the market in the future.

Moreover, we used two text vignettes to reflect two variants of a brief human–robot interaction scenario to manipulate robot autonomy. Text-based scenarios are widely used in psychological research when real-life interactions are either impossible (e.g., in the context of attitudes towards robots [17, 40]) or considered unethical (e.g., in the context of research on sexual violence [21]). Such work emphasizes the utility and validity of a scenario-based experimental manipulation.

We used text-based scenarios in the current research for several methodological reasons: First, at the time the experiment was conducted, an embodied version of the VIVA robot was not yet available. The robot was available only as a concept and pictures, so we used those materials to depict the robot as realistically as possible. Second, we aimed to ensure the best possible trade-of between internal and external validity of the materials. Vignette studies are especially high in internal validity, since most factors are kept constant (i.e., the situation, scenario length, word count) and only the variables of interest (in this case: robot autonomy) are manipulated. However, this is at the expense of external validity [19]. To increase external validity, we followed recommendations by [19] and [1]. That is, we had participants read a short description along with a picture of the robot to facilitate immersion. Further, the scenario that was used to operationalize low autonomy was constructed in accordance with realistic use cases with voice assistants. The scenario depicting a robot high in autonomy, in turn, was constructed to mirror a use case derived from the planned functions and technological possibilities of the VIVA robot, where according to the working definition of autonomy by [6] the robot was capable to sense its environment, plan its behavior and act accordingly.

The two text vignettes were chosen from a pool of 28 scenarios that were carefully pretested in a pilot study with *N* = 97 university students (57 female, 37 male, 3 not specified, $M_{Age}$ = 24.04, $SD_{Age}$ = 3.60) to ensure a successful experimental manipulation of robot autonomy. Each scenario had a similar structure and word count. As a result, we selected two corresponding scenarios. As predicted, in the high autonomy version, the robot was evaluated as significantly more autonomous on a seven-point Likert scale (*M* = 5.47, *SD* = 1.45) than in the low autonomy version (*M* = 3.52, *SD* = 2.08), *t* (96) = 5.34, *p* < 0.001, *d* = 1.09. To illustrate, the scenario that described the VIVA robot low in autonomy translates:

You come home from work and meet VIVA in the living room. As you sit on the sofa and see VIVA, you nod towards VIVA and think about how you would like to spend the evening. You say: ‘VIVA’. VIVA lights up at the sound of the activation-word and asks: ‘How can I help you?’. You say:’ Now I would like to relax. I want to listen to some music. Play an afternoon-playlist.’. ‘Alright, I will play an afternoon-playlist’, VIVA confirms. You listen to the music for a while.

In contrast, the scenario depicting the highly autonomous VIVA robot translates:

You come home from work and meet VIVA in the living room. As you sit on the sofa and see VIVA, you nod towards VIVA and think about how you would like to spend the evening. VIVA notices your nod, nods back and says: ‘Welcome home. How can I help you?’, ‘Now I would like to relax.’, you say. VIVA proposes: ‘Maybe you would like to listen to some music. Do you want me to play an afternoon-playlist?’, and you agree. You listen to the music for a while.

### Measures

Unless otherwise indicated, all responses were assessed via seven-point Likert scales ranging from 1, *completely disagree*, to 7, *completely agree*. Cronbach’s alpha values reflect internal consistencies of the respective scales as measured in the experiment.

#### Robot Evaluation Task

Participants were asked to list positive and negative thoughts or feelings associated with having the social robot VIVA at home. To do so, participants could fill in the blanks for up to 20 entries. The arrangement of the columns (i.e., positive thoughts on the left-hand side and negative thoughts presented on the right or vice versa) was counterbalanced. The open responses produced by participants were analyzed using a Grounded Theory approach [26] to gain insight into the contents of ambivalent attitudes. In the Grounded Theory approach, data are categorized based on empirical results. The emerging categories are re-evaluated concerning their validity in the process, resulting in empirically based concepts that can potentially be used in later hypothesis or theory development. The largest benefit of the Grounded Theory approach is its ecological validity. Rather than using categories derived from the perspective of the researcher, the participants perspective is in the focus, providing valuable insights about real-world settings and attitudes. Furthermore, the approach bears the possibility to discover new categories and attitude aspects that are not yet sufficiently considered in research. The approach is especially useful, when there are not yet enough data to develop a concise hypothesis about a research topic, as is the case for attitudes towards social robots for the home use.

#### Objective Ambivalence

We calculated objective ambivalence, the objective existence of opposing evaluations, as a function of the number of positive and negative entries (cf. [28]). To do so, we used the formula [(P + N)/2]—$|\hbox {P}$–$\hbox {N}|$ with P as the number of positive evaluations and N as the number of negative evaluations [53, 56] resulting in a possible range of – 5 to 10. Higher values indicate higher objective ambivalence.

#### Subjective Ambivalence

Participants were asked to complete a three-item measure of subjective ambivalence adapted from [39]. To do so, we rephrased the items to ask for thoughts and feelings about the VIVA robot. Participants were asked to report feelings of conflict, indecision, and mixed feelings towards the robot ($\alpha$ = .77; e.g., ‘To what degree do you experience mixed feelings towards VIVA?’).

#### Technology Commitment

Since technology commitment has been shown to significantly influence attitudes towards robots (e.g., [40]), this construct was included as a covariate in the main analyses. Thus, participants completed an eight-item questionnaire measuring technology commitment ($\alpha$ = .81) adapted by [41]; original version by [37]. The adaptation consisted of only using the subscales technology acceptance, e.g., ‘I like to use the newest technological devices.’, and technology competence, e.g., ‘I find it difficult to deal with new technology.’ (reverse-coded) and omitting the subscale technology control due to its lack of internal consistency.

#### Additional Variables

*Attention Check, Scenario Perception, and Manipulation Check* To increase data quality, we included two attention checks asking for details of the presented information. Furthermore, we assessed the vividness of participants’ imagination with three items (e.g., ‘It was easy to imagine the scenario.’), the social acceptability of the robot behavior with one item (‘VIVA’s behavior is socially acceptable.’), and administered a one-item manipulation check (‘VIVA is autonomous’.) Moreover, participants reported demographic variables and whether they had already participated in a previous study on the VIVA robot.

*Loneliness* To measure dispositional loneliness, we used the UCLA Loneliness Scale ( $\alpha$ = .72; e.g., ‘I feel isolated from other people.’, ranging from 1, *I never feel that way*, to 4, *I often feel that way * [43], German version by [34]).

*Cognitive and Affective Trust* We assessed the extent to which users trust the robot to function reliably, competently, and predictably (cognitive trust), or to be benevolent and caring (affective trust; cf. [30]). Based on prior work by Bernotat and colleagues [9], participants completed five items concerning cognitive trust ($\alpha$ = .65; e.g., ‘Due to the competence of this robot, I would not hesitate to behave according to its advice.’), and five items measuring affective trust towards the robot (e.g., ‘This robot would only be interested in taking its own advantage.’; $\alpha$ = .67, after the following item was removed:’ This robot would only be interested in personal gain from our relationship.’).

*Robot Likeability* We measured robot likeability using three items ($\alpha$ = .68; e.g. ’VIVA is sympathetic.’, ‘VIVA is polite.”, “VIVA is humble.” [44]). For methodological reasons, we refrained from using popular measurements, such as the Likeability subscale from the Goodspeed Questionnaire [4]. This was due to the fact that its bipolar format (e.g., 1 *dislike* to 5 *like*) simultaneously measures two partly independent constructs in one item (cf. [12]). To illustrate, in the context of such bipolar response format, a medium value of 3 could indicate a neutral attitude or indifference (i.e., the absence of strong positive or negative evaluations). Importantly, however, it could also reflect ambivalence (i.e., the presence of strong positive and negative evaluations, resulting in feelings of conflict and arousal). For a thorough elaboration on this topic see [52].

*Contact Intentions* Because we wanted to explore the cognitive consequences of ambivalence in the context of decision-making, we assessed participants’ contact intentions. Specifically, we asked participants whether they would like to live with the VIVA robot and whether they would like to interact with the robot in a subsequent study. Importantly, we measured participants’ response times to these items as an indicator of evaluative conflict. As preregistered, we excluded trials that deviated to the extent of three standard deviations from the mean. A dichotomous ‘yes-no’ response format was used, since previous research has shown that decisions based on a dichotomous response format yield longer reaction times for ambivalent stimuli compared to univalent stimuli (e.g., [45]).

*Robot Gender* For design evaluation purposes, we included two items on perceived femaleness and maleness of the robot (‘VIVA is female [male]’).

### Procedure

Participants were told they would be asked to evaluate the new social robot VIVA based on a description, an image of the robot, and one text-based example interaction scenario. After the first attention check participants completed the questionnaire measures. They were followed up by a second attention check, the assessment of scenario perception, a manipulation check, and the assessment of demographic data. After giving the opportunity to report the alleged purpose of the experiment, participants were reimbursed and dismissed. Since we did not use any deception and clearly communicated the purpose of the experiment in the beginning, we did not additionally debrief participants in the end. However, participants were given an email-address in case they had any further questions concerning the research.

## Results

### Descriptive Analyses

We used the statistical software R to conduct analyses. As part of the evaluation task, participants reported between two and sixteen thoughts (*M* = 6.64, *SD* = 2.47). Mean objective ambivalence was *M* = 2.08 (*SD* = 1.56; empirical range: − 2.0–6.5) and mean subjective ambivalence was *M* = 4.11 (*SD* = 1.18; empirical range: 1–7), which indicates high ambivalence on absolute levels: According to existing literature on objective and subjective ambivalence, univalence—the absence of ambivalence—is reflected by scores which usually range in the lower tercile of the response scales [46, 56]]. In the present experiment, the lower tercile of the subjective ambivalence scale from 1 to 7 is 2.3 and the lower tercile of the objective ambivalence scale from − 5 to 10 is 0. Participants’ subjective ambivalence towards robots was significantly higher than 2.3, reflecting the lower tercile of the scale (*M* = 4.11, *SD* = 1.18), *t*(162) = 19.64, *p* < .001, *d* = 1.53. The same was the case for objective ambivalence, tested against the lower tercile of 0 (*M* = 2.08, *SD* = 1.56), *t*(162) = 17.04, *p* < .001, *d* = 1.33. This indicated that people generally felt ambivalent towards social robots. Mean technology commitment was moderately high (*M* = 4.81, *SD* = 0.98).

### The Influence of Robot Autonomy on Ambivalence

To test the two main hypotheses that objective (H1) and subjective ambivalence (H2) would be higher in the high autonomy condition compared to the low autonomy condition, we conducted two one-way analyses of covariance (ANCOVA) with robot autonomy as the between-subject condition and technology commitment as a covariate. Contrary to our predictions, there was no significant difference in objective ambivalence towards the robot between the low autonomy condition (*M* = 2.12, *SD* = 1.66) and the high autonomy condition (*M* = 2.02, *SD* = 1.44), *F*(2, 160) = 0.124, *p* = .883 (Hypothesis 1). Further, we did not obtain a significant main effect of autonomy on objective ambivalence either when controlling for technology commitment, *F*(2, 160) = 0.152, *p* = .697, $r^{2} = 0.002$.

Concerning Hypothesis 2, in the high autonomy condition, participants indeed experienced more subjective ambivalence (*M* = 4.27, *SD* = 1.13) than in the low autonomy condition (*M* = 3.96, *SD* = 1.20), *F*(2, 160) = 4.12, *p* = .018. However, this effect turned non-significant when controlling for technology commitment *F*(2, 160) = 3.14, *p* = .078, ${r^{2}} = 0.049$. Technology commitment was a significant predictor of subjective ambivalence (*F*(2,160) = 5.33, *p* = .022). To further investigate the relationship between the two variables, we computed the Pearson correlation coefficient. This analysis revealed that there was a small negative correlation between technology commitment and subjective ambivalence *r*(161) = − 0.17, *p* = .026, indicating a weak relationship. Accordingly, participants higher in technology commitment experienced less attitudinal conflict towards the robot than participants lower in technology commitment.

### Manipulation Check and Scenario Perception

A one-sided t-test revealed that participants perceived the robot to be more autonomous in the high autonomy condition (*M* = 4.55, *SD* = 1.37) than in the low autonomy condition (*M* = 4.18, *SD* = 1.46), *t*(161) = 1.68, *p* = .047,* d* = 0.26. To ensure that the effect of robot autonomy on subjective ambivalence was not caused by systematic differences in participants’ perception of the scenarios, we measured perceived vividness of imagination and social acceptability of the robot behavior. Perceived vividness of participants’ imagination did not significantly differ between the low autonomy condition (*M* = 5.40, *SD* = 1.22) and the high autonomy condition (*M* = 5.49, *SD* = 1.15), *t*(161) = 0.448, *p* = .665. Social acceptability was lower in the low autonomy condition (*M* = 4.54, *SD* = 1.71) than in the high autonomy condition (*M* = 4.99, *SD* = 1.20), *t*(161) = 2.40, *p* = .018, *d* = 0.30.

### Exploratory Analyses Including Additional Variables

In order to explore the nature and structure of attitudes towards robots and to generate a first empirical basis for further theory development, we analyzed the additional variables using t-tests, $\chi 2$- tests, and Pearson correlation analyses. First, we explored the effects of the experimental manipulation on the additional variables to gain further insight into the impact of autonomy on attitudes towards robots. To do so, we conducted ten two-tailed t-tests with adjusted alpha levels according to a Bonferroni correction (*p* < .005) to investigate the effect of robot autonomy on cognitive trust, affective trust, likeability, robot femininity, robot masculinity, response time for the decision to live with the robot, response time for the decision to participate in a future experiment with the robot, number of positive entries in the evaluation task, number of negative entries in the evaluation task, and total number of entries in the evaluation task. People did not seem to trust autonomous robots less, nor did they perceive them as more or less male or female. They also did not show different response times for contact decisions in any condition, nor did they have more positive or more negative entries for any condition, and robot autonomy did not influence the total number of reported entries. However, the robot in the high autonomy condition was rated more likeable than in the low autonomy condition (for inferential statistics, see Table 1).

**Table 1:** Inferential statistics for exploratory analyses as a function of robot autonomy

Variable	$M_{low\,\, autonomy}$ (SD)	$M_{high\,\, autonomy}$ (SD)	*t*	*p*
Cognitive trust	3.52 (0.97)	3.56 (0.94)	0.24	.807
Affective trust	2.65 (1.07)	2.89 (1.24)	1.37	.174
Likeability	4.76 (0.84)	5.18 (0.93)	3.02	.003
Femininity	3.87 (1.64)	4.17 (1.57)	1.18	.241
Masculinity	3.27 (1.52)	3.47 (1.55)	0.85	.398
RT for having the robot at home (in seconds)	3.72 (2.35)	3.93 (2.29)	0.57	.566
RT for participating in study with robot (in seconds)	7.20 (2.79)	7.23 (3.09)	0.07	.947
No. of positive entries	2.99 (1.48)	3.21 (1.24)	1.07	.287
No. of negative entries	3.64 (1.57)	3.45 (1.62)	−0.74	.458
Total no. of thoughts	6.62 (2.64)	6.67 (2.28)	0.11	.912

The fact that participants seemed to like the VIVA robot more when it was described as autonomous was not mirrored in the results on contact intentions. $\chi 2$- tests indicated no difference in the amount of ‘Yes’ versus ‘No’ responses regarding whether participants wanted to live with the robot $\chi 2$(1, *N* = 163) = 0.02, *p* = .880 or meet the robot in another study $\chi ^{2}$(1, *N* = 163) = 3.09, *p* = .079. On a descriptive level, only 43 of 163 participants (26.4%) showed interest in living with the robot, while 121 of 163 participants (74.2%) showed interest in meeting the robot in another study. To test whether those behavioral intentions could be predicted by subjective ambivalence, we conducted two logistic regressions with subjective ambivalence as the predictor. Subjective ambivalence significantly predicted the interest in having a robot at home (*B* = 0.37, *SE* = 0.18, *df* = 1, *p* = .017, *Odd’s* *Ratio* = 1.45). That is, with every increase of 1 in subjective ambivalence, the likeability of choosing ‘No’ increased 1.45 times. However, subjective ambivalence did not predict the interest of participating in another study with the robot (*B* = 0.14, *SE* = 0.16, *df* = 1, *p* = .352, *Odd’s Ratio* = 1.16).

To further explore the relationship between attitudinal ambivalence, robot and user characteristics, we conducted exploratory correlational analyses. Pearson correlation analyses (with an adjusted alpha-level of $\alpha$ < 0.01) did not reveal a statistical relationship between loneliness and objective ambivalence (*r*(161) = .102, *p* = .195) or subjective ambivalence (*r*(161) = .070, *p* = .375). However, people who mentioned more positive entries in the evaluation task felt more affective trust towards the robot (*r*(161) = .268, *p* = .001). This was not the case for cognitive trust (*r*(161) = .036, *p* = .644). Surprisingly, people who reported more negative evaluations did not feel less affective trust (*r*(161) = − .122, *p* = .119), but reported significantly less cognitive trust (*r*(161) = − .281, *p* < .001). This pattern indicated that users might take their positive evaluations into account when judging a robot’s benevolence, but when it comes to judging the robot’s reliability, the users’ negative evaluations seem to be more indicative of their attitude. Further, robot likeability correlated positively with affective trust (*r*(161) = .410, *p* < .001) and cognitive trust (*r*(161) = .253, *p* = .001), and affective and cognitive trust also correlated positively with each other (*r*(161) = .255, *p* < .001). Moreover, highly lonely people seemed to trust the robot more at an affective level (*r*(161) = .276, *p* < .001), but not more on a cognitive level (*r*(161) = − .089, *p* = .257). Finally, reflecting gender stereotypes, people who perceived the robot as more female reported less cognitive trust (*r*(161) = − .244, *p* = .002), but did not differ on affective trust (*r*(161) = .039, *p* = .622). On the other hand, interpreting the robot as more male did not significantly correlate with participants’ affective trust (*r*(161) = − .044, *p* = .576) or cognitive trust (*r*(161) = − .080, *p* = .310).

### Qualitative Analysis

For the qualitative analysis we used all qualitative data from both conditions. Following the Grounded Theory approach [26] we categorized participants’ open responses concerning positive and negative thoughts or feelings associated with having the social robot VIVA at home. When creating categories, we observed many positive and negative evaluations that covered far more topics than those typically addressed in popular measurement instruments to assessing attitudes towards robots (e.g., Negative Attitudes towards Robots Scale (NARS); [38]). Even though instruments such as the NARS [38] cover various domains, these do not sufficiently reflect the concerns voiced by participants in the present research. Instead, such measures tap attitudes linked to concrete interaction aspects (e.g., negative attitude towards the interaction or social influence of robots). Others assess anxiety toward the communication capability of robots, toward behavioural characteristics of the robot, or toward discourse with the robot (see, e.g., Robot Anxiety Scale (RAS); [38]). Thus, based on the qualitative data, new categories for *assets* and *risks* concerning human–robot interaction emerged.

The categories were adjusted in the process, resulting in 17 final categories and two residual categories for positive and negative ambiguous responses or responses that could not be summarized into categories (e.g., “unnecessary”). The categories that emerged from this process concerning *assets* were *assistance, companionship, entertainment, usability, personalization, information, status,* and *surveillance*; and the final categories concerning *risks* were *privacy/data security, isolation, discomfort, trouble, loss of autonomy, realistic threat, inhumanity, abuse,* and *resources* (see Table 2). The residual categories contained responses that did not fit any category. All qualitative data were assigned to one of those categories by two independent raters to ensure the validity of the entries’ assignment to categories. The interrater reliability was very high for both positive (*Kappa* = 0.91, *p* < .001) and negative entries (*Kappa* = 0.93, *p* < .001). As depicted in Table 2, we analyzed the occurrence of the categories in absolute terms (frequency of mentions) as well as relative terms (percentage of mentions). Regarding the positive entries, most aspects concerned assistance, companionship, and usability; regarding the negative entries, the categories privacy/data security, loss of autonomy and technological trouble were named most frequently.

**Table 2:** Categories, original example entries, frequencies, and percentages for qualitative entries regarding users’ positive and negative evaluations of having a social robot at home

Topic	Example entry	Frequency	%
*Positive*			
Assistance	‘Help in everyday life’	241	37.7
Companionship	‘Not feeling lonely’	92	14.2
Usability	‘Useable for people of all ages’	44	6.8
Entertainment	‘Funny’	40	6.2
Information	‘Quick access to information’	31	4.8
Status	‘To be up to date with technology’	16	2.5
Personalization	‘Adaptable to the person’	9	1.4
Surveillance	‘Somebody watches the home’	5	0.8
Other	‘Like a pet’	27	4.2
*Negative*			
Privacy/Data Security	‘Risk of monitoring/access to data by third parties’	143	22.1
Loss of Autonomy	‘Depending on the robot’	81	12.5
Technological trouble	‘Technology could be prone to malfunctions’	77	11.9
Resources	‘High power consumption’	64	9.9
Inhumanity	‘No replacement for human interaction’	54	8.4
Discomfort	‘Feeling of being watched’	41	6.3
Isolation	‘Neglecting social contacts’	33	5.1
Abuse	‘Could be hacked’	31	4.8
Realistic Threat	‘Danger of becoming autonomous’	22	3.4
Other	‘Unnecessary’	30	4.6

$$N_{entries} = 827$$

## Discussion

Research on attitudes towards robots in general has often described these attitudes as neutral [17, 40]. However, as has become clear from previous research, neutral ratings might mask underlying ambivalent attitudes (cf. [46, 52]). Ambivalence towards robots caused by opposing evaluations, and the specific content of those evaluations has not yet been investigated in detail. Therefore, the present research provides valuable insights for both social robotics and social psychological research.

Our contribution builds on basic research on attitudes towards robots by taking into account social psychological methods and findings (i.e., research on the ambivalent nature of attitudes). We have explored the external validity of this theorizing and the respective methods used to study ambivalence by empirically testing and validating them in the domain of robots. To address these research issues, we conducted the current experiment. Here, we investigated the influence of robot autonomy on ambivalent attitudes towards the VIVA robot and analyzed the contents of those attitudes using a qualitative and a quantitative approach. People reported numerous positive and negative evaluations concerning social robots, and a feeling of being ‘torn’ between two sides of an attitude, especially towards the more autonomous social robot. Attitudes in both the low autonomy and the high autonomy condition were highly ambivalent which indicated that robots evoked high levels of attitudinal conflict in general, and the robot’s autonomy level did not have a large additional influence on ambivalence. It is possible that individual differences play a larger role in the experience and resolution of ambivalence than robot-related variables.

Contrary to our hypotheses, objective and subjective ambivalence were not higher in the high autonomy condition compared to the low autonomy condition, when controlling for technology commitment. In this experiment, our measure of objective ambivalence might not have been sensitive enough. To assess objective ambivalence, we had relied on the formula [(P + N)/2] – $|\hbox {P}$ – $\hbox {N}|$ [5]. According to this formula, a user naming five very strong positive points and five very weak negative points would receive the same objective ambivalence score as a user naming five strong positive and five strong negative points, despite potentially experiencing less ambivalence. Future research could additionally ask participants to rate the subjective significance of their entries in the evaluation task, to estimate the magnitude of objective ambivalence more precisely.

Interestingly, our results showed that subjective ambivalence was higher in the high autonomy condition than in the low autonomy condition when refraining from controlling for technology commitment. However, when controlling for technology commitment, the influence of robot autonomy on subjective ambivalence turned non-significant. Apparently, technology commitment did not only influence the formation and resolution of ambivalence in attitudes towards robots: Importantly, correlational analyses indicated that users high in technology commitment experience less ambivalence overall. That is, people high in technology commitment might have a more realistic view of the possibilities and challenges of robots and do not feel conflicted and ‘torn’ between positive and negative evaluations as people low in technology commitment. In the current student sample, mean technology commitment was relatively high. One possible reason for the fact that there was no significant effect of autonomy on ambivalence when controlling for technology commitment could be that robot autonomy might primarily influence attitudes of people low in technology commitment. That is, people low in technology commitment might be more sceptical towards robots in general, while at the same time overestimating robotic abilities, resulting in highly ambivalent attitudes. It should be noted that there was only a small effect of the experimental manipulation on perceived autonomy. Possibly, a more pronounced manipulation of robot autonomy could produce larger differences in ambivalent attitudes.

Considering scenario perception, our manipulation check indicated that participants perceived the robot to be more autonomous in the high autonomy condition compared to the low autonomy condition. This indicates that participants read the instruction conscientiously and the manipulation was effective. While perceived vividness did not differ between conditions, social acceptability was lower in the low autonomy condition compared to the high autonomy condition. This might be due to the fact that the robot in the low autonomy condition did not show any independent behavior that could be interpreted as highly socially acceptable but rather followed instructions. Despite differing between conditions, these results indicate that potential differences in the social acceptability of the scenarios do not provide an alternative explanation for the difference in subjective ambivalence between conditions. We would have expected higher ambivalence for low social acceptability, and not for higher social acceptability, since inappropriate behavior seems more likely to cause feelings of conflict than appropriate behavior.

Exploratory analyses revealed that another important dispositional variable to be considered in research on attitudes towards robots might be chronic loneliness, as loneliness was positively correlated with affective trust, but not with cognitive trust. Previous research indicated that loneliness is associated with anthropomorphism concerning robots [20]. Here, a tendency to anthropomorphise the robot might have led to a higher faith in the robot’s benevolence (i.e., cognitive trust), while it did not affect the faith in the robots functionality (i.e., affective trust) since it is independent of anthropomorphism. This underlines the notion that loneliness not only influences social interaction, but also the perception and evaluation of potential interaction partners before the interaction. These results should be interpreted with caution due to the lack of internal consistency for the likeability and trust scales and the exploratory nature of results and should be readdressed in future research. Loneliness did not correlate significantly with objective or subjective ambivalence. Thus, loneliness did not seem to attenuate evaluative conflict, but rather appeared to influence attitudes towards the robot indirectly by prompting a perception of the robot as more benevolent.

Further exploratory analyses showed that in the high autonomy condition, the robot was perceived as more likeable than in the low autonomy condition. This might indicate positive attitudes towards highly autonomous robots, as their behavior is perceived as more socially acceptable and less annoying. These results suggest that highly autonomous robots might be more likeable as well as being perceived as more ambivalent. Despite ambivalence being an aversive state, ambivalence is likewise associated with important benefits. That is, the experience of ambivalence leads to more thorough information processing and less susceptibility to bias [48]. This is due to the fact that individuals who hold highly ambivalent attitudes are potentially more interested in further information on the robot in order to resolve their attitudinal conflict. However, these exploratory results should be interpreted with caution since the scale for likeability lacked internal consistency.

Concerning behavioral intentions, $\chi$2-tests did not indicate a higher interest in living with the robot or meeting the robot in another study depending on the condition. It is interesting that overall, most participants reported intentions to meet the robot, while only a small part of participants was interested to have the robot at home. However, ambivalence might influence behavioral intentions: Subjective ambivalence significantly predicted the interest in having a robot at home, but not the interest of meeting the robot in another study. This might indicate that ambivalent users are equally interested in learning more about robots than univalent users, however, they might to want to avoid integrating them in their everyday life. This apparent gap between a general interest in robots and a reluctance to welcome them into users’ homes might be interpreted with the use of the qualitative data.

Crucially, thanks to the multi-method approach, we were able to gain insights into the specific contents of robot-related evaluations. By means of the qualitative data analysis, we were able to identify aspects that have not completely been theoretically considered in the development of popular instruments to measure those attitudes. Interestingly, many people reported fear of losing autonomy or of being socially isolated because of using a social robot, while at the same time, many people voiced the hope that the VIVA robot could serve as a social companion, preventing them from feeling lonely. Another conflict that emerged from qualitative data analyses reflects participants’ high hopes for the robot’s usefulness, while they simultaneously appear worried with regard to security and privacy violations associated with social robots. Those violations might go hand in hand with the robot’s usability, as a robot needs to be more autonomous and integrate more and private information (e.g., save the users face, social network, tasks, routines) in order to be highly useful (e.g., remember people, things and tasks, recommend activities, solve problems). Users seemed aware of ethical problems in the use of social robots, as many categories corresponded to current analyses on ethical challenges concerning social robots, e.g., privacy, data protection and safety [33].

Furthermore, the qualitative data might provide possible explanations for the gap between a general interest in robots and a reluctance to welcome them into users’ homes. The negative evaluations of the robot (e.g., social isolation, data security issues, loss of autonomy, technological trouble) might be specifically weighty when living with the robot, while the positive evaluations might be experienced already in a short contact (e.g., help with a task, rewarding social interaction, good usability). The qualitative results showed that potential users’ evaluations of the robot are manifold and covered many different domains, ranging from topics revolving around everyday use, e.g., assistance in everyday life, topics addressing existential issues and fears, e.g., feeling threatened by robots. More research is needed to investigate the contents of ambivalent attitudes more concretely in order to identify specific strategies to reduce ambivalence and further robot acceptance.

### Strengths and Limitations

To our knowledge, the current experiment was the first to investigate the influence of robot autonomy on attitudinal ambivalence towards a social robot. We conducted a preregistered experiment based on a realistic use case. The use of text-based scenarios represents a strength as well as a weakness of the current research: The strength of vignette studies is their high internal validity since their use allows to manipulate key concepts while keeping the other information constant for all participants. Further research is planned with the VIVA robot in real-life interactions, as soon as laboratory experiments are permitted again after the Covid-19 pandemic. We extended our quantitative research by qualitative methods and gained insight into the contents of potential users’ evaluations of robots. Furthermore, we identified a possible mechanism for the influence of technology commitment on attitudes towards robots, namely a role in the emergence and resolution of attitudinal conflict towards robots: Correlational analyses indicated that users high in technology commitment experience less ambivalence towards robots overall. The relationship between technology commitment and ambivalence should be investigated further using response-time based methods, e.g. mouse tracking. High technology commitment might lead to quicker decision making concerning robots or a quicker resolution of attitudinal conflict, as has been shown for self-control concerning ambivalence in general [47].

Another strength of this study concerns the application of social psychological theorizing on attitudinal ambivalence to a human–robot interaction scenario. Our findings point out possibilities to reduce ambivalence in attitudes towards robots in the home context as well as in research scenarios. This might be achieved by reducing user concerns regarding their data privacy and ensuring the highest cyber-security standards or implementing full offline functionality, while at the same time accentuating a robot’s positive features, such as its usefulness and its social competencies. We suggest that by fostering a decrease of negative evaluations and by contributing to an increase of positive evaluations, ambivalent attitudes towards robots are likely to become univalent, positive attitudes. This certainly calls for further empirical research, also using longitudinal approaches. We hereby provide a study design that may be tested in future experiments, in order to investigate ambivalence towards autonomous robots in real-life human–robot interactions

The present results revealed that the psychometric quality of the exploratory variables (e.g., robot likeability and trust) represents a weakness of the current research. The low levels of internal consistency call for further adaptations and validation of popular measures to assess attitudes towards robots. Moreover, one could draw from the literature in social psychology to adapt measurements on attitudes. One possibility could be to use validated scales from social psychological research (e.g., concerning trust) and transfer them to the robotics context and extend them by behavioral (e.g., response time based) measures. Response time based methods, e.g., the implicit association test [27] or mouse tracking [23] have been administered to complement attitude research by measuring implicit attitudes that participants are not willing or able to disclose in self-report measures. Furthermore, in our experiment, autonomy did not have a large impact on ambivalence in attitudes towards robots. Prospective research should investigate specific factors related to autonomous robots (e.g., voice control, face recognition, privacy settings) that influence attitudinal ambivalence towards robots, while taking technology commitment as a moderating factor into account.

Participants’ judgments were based on limited information only and might have been biased by participants’ preexisting attitudes toward robots in general. It is plausible that our results replicate with other social robots that feature childlike anthropomorphic designs, such as Nao, Pepper, or FloKa (cf. [7]). To test the generalizability of our results empirically, prospective research should investigate ambivalence using more diverse samples, by deploying different types of robots in text-based scenarios, pictures, videos, or actual human–robot interactions. It is especially important to replicate our results in human–robot interaction experiments, since it is likely that results from vignette studies might differ from results from real-life interaction studies. Due to the multi-modal nature of a real-life encounter with a robot, participants might report different affective and cognitive responses, compared to an imagined situation. Nevertheless, investigating prospective attitudes towards robots is essential, since attitudes guide behavioral intentions and behavior [10], e.g., contact intentions, interest in purchasing a robot, or readiness to participate in robot-related research. Prospective research might investigate such behavioral consequences of attitudinal ambivalence in detail.

Furthermore, the current sample consisted of university students. It is likely that certain characteristics, (e.g., students’ high technology commitment) might have influenced results. Sample characteristics may be especially important in the field of social robotics and results might differ depending on the age group, i.e., young adults seem to evaluate robots more positively in general [5]. Yet, our results are relevant for the development of social robots such as VIVA, particularly, because young adults represent a key potential end user group. However, future studies should investigate whether the effect of robot autonomy on ambivalence replicates across other age groups and in the general population. We would speculate that effects would even be more pronounced compared to the current results. This is due to the fact that robot autonomy might specifically influence attitudes of people low in technology commitment. In fact, people who share this disposition might have been underrepresented in the current sample. Another interesting future investigation might be to employ the Grounded Theory approach in research on attitudes towards social robots with elderly people in order to explore differences in results across various age cohorts.

## Conclusion

In sum, results based on qualitative and quantitative data from a student sample have revealed that first, attitudes towards autonomous robots are ambivalent and second, they might be prone to influences by individual dispositions, such as an individual’s technology commitment. Ambivalence seems to be an important factor in attitudes towards robots that affected behavioral intentions, as indicated by exploratory results. Above and beyond, we expanded the literature on attitudinal ambivalence by extending its scope to social robots and by testing the notion in the applied domain, featuring an actual use case. We demonstrated that ambivalence research may benefit from a combination of traditional quantitative ambivalence measures with qualitative analyses in order to gain insights into the specific attitude contents, depending on the research topic. Potential robot users (i.e., university students) reported various positive and negative evaluations about robots, especially the hope of robots being useful and being social companions on one hand, and privacy and security concerns, and a fear of losing autonomy, on the other hand. Our results support the idea that people, in fact, view robots as a threat [57]. However, it seems like this perception of robot-related threat served to express their concerns regarding autonomy loss and data security rather than reflecting the experience of physical threat by a robot. Future research should adapt reliable measurement methods from social psychological research to complement currently available measurement instruments to assess robot-related attitudes. The insights from qualitative data bear the potential to improve attitudes towards robots in various contexts by directly addressing the users’ hopes and fears. Taken together, potential end users do not seem to be afraid of a robot apocalypse—they are rather quite aware of the many challenges that come with the use of robots, while at the same time holding high expectations regarding their benefits and use.
